# Effect of high normal body mass index and its trajectory on risk of new-onset hypertension among Chinese adults: a national prospective cohort study

**DOI:** 10.3389/fcvm.2025.1684124

**Published:** 2025-11-21

**Authors:** Hongxue Yu, Qi Gao, Yuxin Lin, Fan Luo, Pingping Li, Yuping Zhang, Jiao Liu, Ruqi Xu, Yanqin Li, Licong Su

**Affiliations:** 1 Division of Nephrology, Nanfang Hospital, Southern Medical University, Guangzhou, China; 2 National Clinical Research Center for Kidney Disease, Guangzhou, China; 3 State Key Laboratory of Organ Failure Research, Nanfang Hospital, Southern Medical University, Guangzhou, China; 4 Guangdong Provincial Institute of Nephrology, Guangzhou, China; 5 Guangdong Provincial Key Laboratory of Renal Failure Research, Nanfang Hospital, Southern Medical University, Guangzhou, China; 6 Department of Nephrology, Wuhan Fourth Hospital, Wuhan, China; 7 Wuhan Clinical Research Center for Metabolic Chronic Kidney Disease, Wuhan, China; 8 Division of Nephrology, People's Hospital of Yangjiang, Yangjiang, China

**Keywords:** China health and nutrition survey, high normal BMI, BMI trajectories, new-onset hypertension, prospective cohort study

## Abstract

**Background:**

Obesity or overweight is well known to be a strong risk factor for the development of hypertension. However, the association of body mass index (BMI) within the normal range and its trajectory with new-onset hypertension in adults remains incompletely understood.

**Methods:**

A total of 9,583 participants with normal BMI, who were without hypertension at baseline and underwent at least 2 rounds of visits from 1989 to 2015 in the China Health and Nutrition Survey were enrolled in this study. Multivariate Cox hazard regression models and restricted cubic spline were used to explore the relationship between BMI and new-onset hypertension. The latent class growth mixed model (LCGMM) was used to identify different trajectory patterns of normal BMI, including stable, increasing and fluctuating groups.

**Results:**

Of 9,583 eligible participants, 3,025 (31.6%) participants developed new-onset hypertension during a median (interquartile range, IQR) follow-up duration of 8.9 (4.1, 15.1) years. After adjusting for confounders, the Cox model showed that high BMI levels in normal range were significantly associated with increased risks of new-onset hypertension [adjusted hazard ratio (aHR), 1.11, 95% confidence interval (CI), 1.01–1.21, tertile 2 (T2)]; aHR, 1.38, 95% CI, 1.26–1.51, tertile 3 [T3]) compared with tertile 1 (T1). As a continuous variable, for per 1.0 kg/m^2^ increment in BMI, there was a 10% increment in the risk of hypertension (aHR, 1.10; 95% CI, 1.07–1.12). The associations were consistent in various subgroups and sensitivity analyses. Compared to stable group, the increasing and fluctuating trajectories were also significantly associated with hypertension, respectively (aHR, 1.19, 95% CI, 1.03–1.38, the increasing group; aHR, 1.26, 95% CI, 1.07–1.48, the fluctuating group).

**Conclusion:**

The high normal BMI was significantly associated with an increased risk of new-onset hypertension among Chinese adults, whether increasing or fluctuating trajectory. Our findings suggest that maintaining a relatively low BMI level with stable trajectory within the normal range might be effective for the primary prevention of hypertension.

## 1 Introduction

Hypertension, especially high systolic blood pressure is a significant contributor to morbidity and mortality associated with cardiovascular diseases, kidney diseases, and other noncommunicable diseases globally (1). It accounted for 10.4 million deaths with a 95% uncertainty interval (UI) from 9.39 to 11.5 and 218 million (95% UI, 198–237) disability-adjusted life-years in 2017 (1, 2). In 2019, there were 1.28 billion cases aged 30–79 years of hypertension globally, with a doubled number since 1990 (3). Also, the prevalence of hypertension has increased in mainland China during 2014–2017, with 44.7% of the population aged 35–75 years suffering from hypertension. However, the percentages of control [median (interquartile range, IQR), 30.1% (30.0–30.2)] and the achievement of blood pressure goals [median (IQR), 7.2% (7.1–7.2)] remained low in China (4). Therefore, it is crucial to identify and manage modifiable risk factors to prevent the incidence of hypertension.

Obesity and overweight, as defined by body mass index (BMI), are believed to influence the incidence and development of hypertension (5–10). While some previous studies have found that high BMI was associated with increased risks of new-onset hypertension (11–18), these studies mainly focused on whole range of BMI. Evidence remains limited regarding the effects of BMI within the normal range on the risk of hypertension in Chinese adults. Only two previous studies have found that high BMI within the normal range was associated with an increased risk of hypertension in children and adolescents (19, 20). Thus, it remains unclear whether BMI and its trajectory exert an effect within the normal interval on the risk of incident hypertension in adults.

In this study, we aimed to evaluate the potential association between BMI as well as its trajectory and incident hypertension in Chinese adults with normal weight based on data from the China Health and Nutrition Survey (CHNS) (21).

## 2 Methods

### 2.1 Study design, population, and data sources

The study population was drawn from the CHNS cohort, which has been previously described (21). The data, as well as study materials that support the findings of this study, are available on the CHNS website (http://www.cpc.unc.edu/projects/china). Briefly, the CHNS is an ongoing, large-scale, prospective, multistage cohort established in 1989 among the Chinese population. By 2015, the longitudinal study had enrolled 42,829 people from 388 communities in 15 provinces and autonomous regions. The provinces included in the CHNS constituted 47% of China's population (22). To date, 10 follow-up waves (in 1989, 1991, 1993, 1997, 2000, 2004, 2006, 2009, 2011, and 2015) have been completed. At each follow-up survey round, information on demographics, socioeconomics, diet, lifestyle habits (including smoking and alcohol consumption), and medical health was recorded by trained personnel.

**Figure 1:**
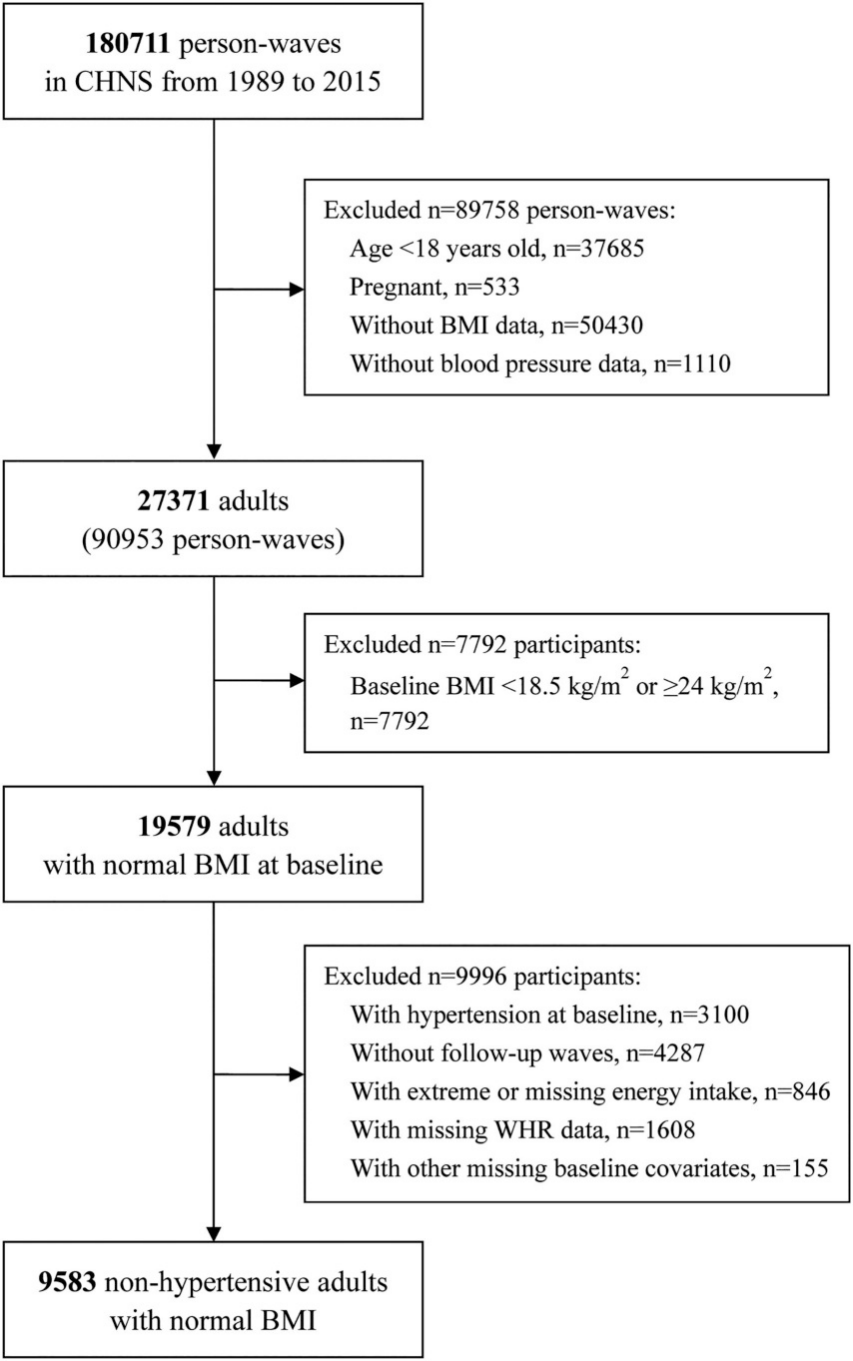
Flowchart of participants selection. CHNS, China Health and Nutrition Survey; BMI, body mass index.

The baseline was defined as the first visit with normal BMI. Among 42,829 participants with 180,711 person-waves in CHNS, we first excluded ineligible waves: participants were younger than 18 years (*n* = 37,685 waves), pregnant (*n* = 533 waves), lacking BMI data (*n* = 50,430 waves), or without blood pressure data (*n* = 1,110 waves). Then, we excluded participants with abnormal BMI at baseline based on the Chinese consensus (<18.5 kg/m^2^ or ≥24 kg/m^2^) (23–25) (*n* = 7,792). Of the 19,579 adults with normal BMI, we further excluded participants with hypertension at baseline (*n* = 3,100), with no follow-up visits (*n* = 4,287), who had extreme dietary energy intake (<800 or >8,000 kcal/d for male and <600 or >6,000 kcal/d for female) (*n* = 846), without waist to hip ratio (WHR) data (*n* = 1,608), or without other baseline covariates (*n* = 155). Finally, a total of 9,583 non-hypertensive participants with normal BMI were included in the analysis (Figure 1).

The study was approved by the institutional review committees of the University of North Carolina at Chapel Hill, the National Institute of Nutrition and Food Safety, and the Chinese Center for Disease Control and Prevention. Each participant provided their written informed consent. The study complies with the Declaration of Helsinki and the STROBE (Strengthening the Reporting of Observational Studies in Epidemiology) statement (26) was followed in the reporting of this study.

### 2.2 Covariates

Demographic data including age, sex (male or female), smoking status (never, ever, or current), drinking status namely alcohol assumption (never, ever, or current), region (north or south), urban residence (yes or no), education level (illiteracy, primary school, middle school, or high school or above), and occupation (farmer, worker, unemployed, or others) were obtained from standard questionnaires. WHR was calculated as waist circumference (m)/hip circumference (m). Individual dietary intakes including energy, carbohydrate, fat, and protein intakes calculated using the Food Composition Tables (FCT) for China were included in our study. Diabetes mellitus (DM) was defined as self-reported physician-diagnosed diabetes, taking oral hypoglycemic medications or insulin injection (21, 22, 27–29).

Standardized seated blood pressure on the same arm of every participant was measured three times using a mercury manometer by trained research staff after participants had rested for at least 5 min. The average values of three systolic blood pressure (SBP) and diastolic blood pressure (DBP) measurements were calculated and used in our study (27–29).

### 2.3 Exposure

BMI was calculated as weight (kg)/height (m) squared (23). The study population was divided into three groups based on the tertiles of baseline BMI or three trajectories based on the best fitting model.

### 2.4 Outcome

The study outcome was new-onset hypertension, defined as mean SBP ≥140 mmHg and/or mean DBP ≥90 mmHg, or a physician hypertension diagnosis, or undergoing treatments for hypertension during follow-up in accordance with criteria of the World Health Organization (WHO).

### 2.5 Statistical analyses

Continuous variables were presented as the mean ± standard deviation (SD) for normally distributed data compared using One-Way ANOVA or a median (interquartile range, IQR) for data that were not normally distributed compared using Kruskal–Wallis test. Categorical data were presented as a number (percentage) and compared using Pearson *χ*^2^ test.

The study population was divided into three groups based on the tertiles of baseline BMI. The first wave of eligible visits with normal BMI was considered baseline. The follow-up period started from the baseline to the date of the first occurrence of an outcome or the latest survey round (in 2015) or lost to follow-up, which came first. The new-onset hypertension incidence rate was expressed as per 1,000 person-years. The univariate and multivariate Cox proportional hazard regression models were conducted to identify the association of BMI with new-onset hypertension, and hazard ratios (HRs) were expressed with their 95% confidence intervals (95% CI). Model 1 adjusted for age, sex, WHR, baseline SBP and DBP, and smoking/drinking status. Model 2 adjusted for the variables included in Model 1, as well as urban residence, region, education level, occupation, dietary intakes, and presence of DM. In addition, the latent class growth mixed model (LCGMM) was used to identify different trajectory patterns of normal BMI in participants who were 18–64 years with at least three successive normal BMI. The latent class trajectories of BMI were specified as a function of age. The proportional hazards assumption was tested by plotting Schoenfeld residuals against time, followed by a visual inspection for uniformity. The variance inflation factor (VIF) (30) for all predictors in the models was less than 5, indicating the absence of significant multicollinearity. We also performed restricted cubic spline (RCS) Cox regression, with four knots, to test for linearity and characterize level-response relationships between BMI and new-onset hypertension.

### 2.6 Subgroup analyses

The possible modifications of the association between BMI in normal range and new-onset hypertension were performed in several subgroups. Participants were stratified by age (<65 vs. ≥65 years), sex (female vs. male), WHR [≤0.80 (median) vs. >0.80], presence of DM (no vs. yes), urban residence (no vs. yes), region (north vs. south), and presence of pre-hypertension defined as SBP 120–139 mmHg, and/or DBP 80–89 mmHg without antihypertensive medication (31) (no vs. yes). We included an interaction term in the model for each analysis to assess effect measure modification.

### 2.7 Sensitivity analyses

The robustness of the study results was further verified by various sensitivity analyses. Firstly, given that the exact time-point of outcome occurred is difficult to capture, we fitted a Cox model using interval-censoring methods (30) to assess whether results were affected. Secondly, we excluded information on hypertension obtained through questionnaires including the presence or absence of hypertension and the use of antihypertensive medication, since there might be a recall bias. Thirdly, we modified the diagnostic thresholds in accordance with American College of Cardiology (ACC) and American Heart Association (AHA) guidelines (32). In this part, hypertension was redefined as an average SBP ≥130 mmHg and/or an average DBP ≥80 mmHg, a physician hypertension diagnosis, or taking antihypertensive medication. In addition, we redefined the normal-weight participants as a BMI between 18.5 kg/m^2^ and 24.9 kg/m^2^ based on the criterion of WHO (33). Also, we re-analyzed the data in participants who had normal BMI at all visits. Then, we re-identified BMI trajectories in participants aged ≥18 years with at least three successive normal BMI. Finally, given that participants were enrolled in different time, we further adjusted the calendar year of enrollment in Cox models.

All the above statistical analyses were performed using the R software (version 4.1.2; http://www.r-project.org/). A two-sided *P* < 0.05 was considered to be statistically significant in all analyses.

## 3 Results

### 3.1 Study population and baseline characteristics

The flowchart of the study population selection is shown in Figure 1. Of the 9,583 participants selected for analysis [of whom 4,560 were male (47.6%)], the median (IQR) age was 36.0 (27.0, 47.0) years, and the median (IQR) baseline SBP and DBP were 110.0 (103.0, 120.0) mmHg and 73.0 (69.0, 80.0) mmHg, respectively (Table 1).

**Table 1:** Baseline characteristics of participants stratified by tertiles of BMI.

Characteristics	Overall	Tertiles of BMI			
		T1 (18.5–20.4)	T2 (>20.4–22.0)	T3 (>22.0–<24.0)	*P* value
*n*	9,583	3,291	3,217	3,075	
BMI, kg/m^2^	21.2 [20.0, 22.5]	19.6 [19.1, 20.0]	21.2 [20.8, 21.6]	22.9 [22.5, 23.4]	**<0**.**001**
Age, years	36.0 [27.0, 47.0]	34.0 [25.0, 46.0]	36.0 [27.0, 46.0]	39.0 [30.0, 49.0]	**<0**.**001**
Male (%)	4,560 (47.6)	1,572 (47.8)	1,551 (48.2)	1,437 (46.7)	0.485
SBP, mmHg	110.0 [103.0, 120.0]	110.0 [100.0, 119.0]	110.0 [104.0, 120.0]	114.0 [107.0, 120.0]	**<0**.**001**
DBP, mmHg	73.0 [69.0, 80.0]	70.0 [66.0, 79.0]	73.0 [69.0, 80.0]	75.0 [70.0, 80.0]	**<0**.**001**
WHR	0.8 [0.8, 0.9]	0.8 [0.8, 0.9]	0.8 [0.8, 0.9]	0.9 [0.8, 0.9]	**<0**.**001**
Smoking, *n* (%)					0.311
never	6,536 (68.2)	2,231 (67.8)	2,163 (67.2)	2,142 (69.7)	
ever	123 (1.3)	41 (1.2)	43 (1.3)	39 (1.3)	
current	2,924 (30.5)	1,019 (31.0)	1,011 (31.4)	894 (29.1)	
Drinking, *n* (%)					**0**.**003**
never	6,315 (65.9)	2,250 (68.4)	2,067 (64.3)	1,998 (65.0)	
ever	113 (1.2)	29 (0.9)	46 (1.4)	38 (1.2)	
current	3,155 (32.9)	1,012 (30.8)	1,104 (34.3)	1,039 (33.8)	
Urban residence, *n* (%)	3,115 (32.5)	948 (28.8)	957 (29.7)	1,210 (39.3)	**<0**.**001**
Region^a^, *n* (%)					**<0**.**001**
North	3,432 (35.8)	968 (29.4)	1,191 (37.0)	1,273 (41.4)	
South	6,151 (64.2)	2,323 (70.6)	2,026 (63.0)	1,802 (58.6)	
Education, *n* (%)					**0**.**041**
Illiteracy	1,844 (19.2)	655 (19.9)	611 (19.0)	578 (18.8)	
Primary school	1,904 (19.9)	671 (20.4)	653 (20.3)	580 (18.9)	
Middle school	4,619 (48.2)	1,568 (47.6)	1,573 (48.9)	1,478 (48.1)	
High school or above	1,216 (12.7)	397 (12.1)	380 (11.8)	439 (14.3)	
Occupation, *n* (%)					**<0**.**001**
Farmer	3,802 (39.7)	1,429 (43.4)	1,340 (41.7)	1,033 (33.6)	
Worker	3,157 (32.9)	1,008 (30.6)	1,046 (32.5)	1,103 (35.9)	
Unemployed	2,207 (23.0)	718 (21.8)	701 (21.8)	788 (25.6)	
Others	417 (4.4)	136 (4.1)	130 (4.0)	151 (4.9)	
Dietary intake, g/d					
Energy	2,259.1 [1,825.7, 2,709.9]	2,262.0 [1,834.0, 2,708.9]	2,269.6 [1,837.6, 2,737.4]	2,233.0 [1,798.9, 2,685.2]	**0**.**026**
Fat	60.9 [41.1, 85.9]	59.2 [40.0, 83.5]	60.4 [41.1, 85.0]	64.2 [42.6, 89.0]	**<0**.**001**
Carbohydrate	338.0 [251.0, 429.8]	345.5 [258.4, 437.4]	345.2 [256.3, 432.9]	325.4 [241.0, 417.0]	**<0**.**001**
Protein	67.1 [54.1, 83.3]	66.8 [54.0, 82.3]	67.0 [54.2, 83.1]	67.4 [54.2, 84.3]	0.183
Diabetes mellitus, *n* (%)	96 (1.0)	17 (0.5)	25 (0.8)	54 (1.8)	**<0**.**001**

Results are expressed as median [interquartile range] or number (percentage).

n, number; T, tertile; BMI, body mass index; SBP, systolic blood pressure; DBP, diastolic blood pressure; WHR, waist to hip ratio.

a Region was divided into north (Heilongjiang, Liaoning, Shandong, and Henan), and south (Jiangsu, Hubei, Hunan, Guizhou, and Guangxi) based on the Qinling Mountains-Huaihe River Line.

The demographic and baseline characteristics of the included participants stratified by the tertiles of BMI are summarized in Table 1. The median (IQR) BMI was 21.2 (20.0, 22.5) kg/m^2^. In general, compared to the lowest tertile group, those in the high tertile were older, and more likely to live in northern China and in urban residence; they had higher values of SBP, DBP, WHR, total fat intakes, and a higher prevalence of DM (all *P* < 0.05). In addition, there were no significant differences in sex and smoking status in these groups (all *P* > 0.05). The baseline characteristics of participants stratified by outcome were compared in Supplementary Table S1.

### 3.2 Association between BMI and new-onset hypertension

**Figure 2:**
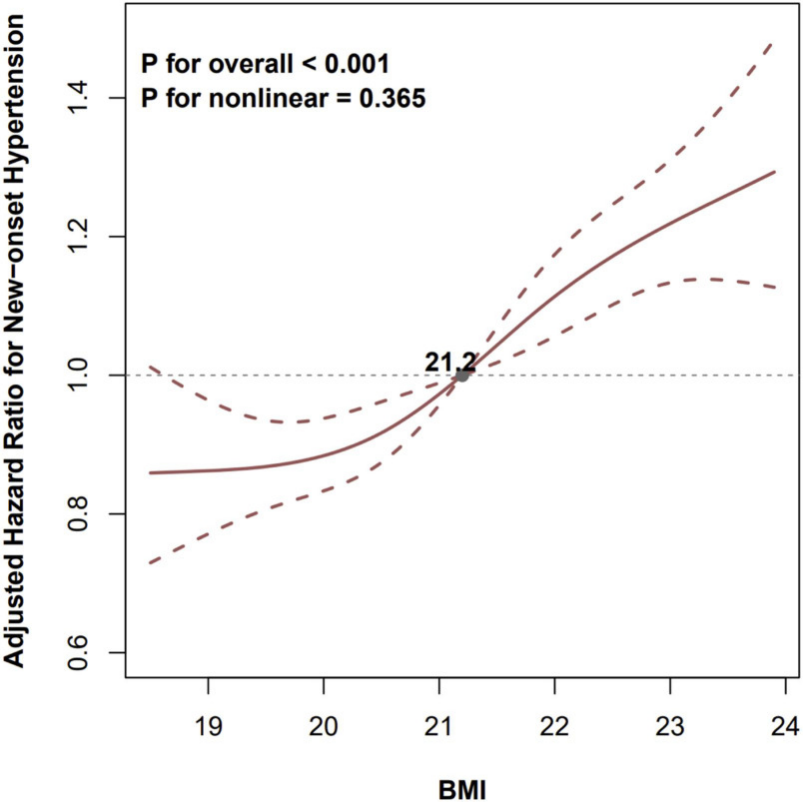
Levels of BMI and the risk of new-onset hypertension. BMI, body mass index; WHR, waist to hip ratio; SBP, systolic blood pressure; DBP, diastolic blood pressure. The gray dot indicates the value of BMI with the hazard ratio of 1.0 for new-onset hypertension. The model was adjusted for sex, age, WHR, SBP, DBP, smoking, drinking, region, urban residence, education, occupation, dietary intake of fat, protein and carbohydrate, and diabetes mellitus.

A total of 3,025 (31.6%) participants developed new-onset hypertension during a median (IQR) follow-up duration of 8.9 (4.1, 15.1) years. The incidences of new-onset hypertension were 25.64%, 30.06%, and 40.20% across BMI tertiles, from tertile 1 (T1) to tertile 3 (T3), respectively. After adjusting for confounders, the Cox model showed that high levels of BMI were significantly associated with an increased risk of new-onset hypertension [adjusted hazard ratio (aHR), 1.11, 95% confidence interval (CI), 1.01–1.21, tertile 2 (T2); aHR, 1.38, 95% CI, 1.26–1.51, T3] compared with T1 group. We also found that this risk increased progressively as the BMI increased (P for trend <0.001). Consistently with the above analysis, when BMI was treated as a continuous variable, for per 1.0 kg/m^2^ increment in BMI, there was a 10% increment in the risk of new-onset hypertension (aHR, 1.10; 95% CI, 1.07–1.12) (Table 2). The dose-response curve indicated a positive, linear association between BMI and the risk of new-onset hypertension (Figure 2).

**Table 2:** The association of BMI with new-onset hypertension.

BMI (kg/m^2^)	Total patients	No. of events (incident rate^a^)	Crude model		Model 1		Model 2	
			HR (95% CI)	*P* value	HR (95% CI)	*P* value	HR (95% CI)	*P* value
Tertiles								
T1 (18.5–20.4)	3,291	904 (25.64)	Reference		Reference		Reference	
T2 (>20.4–22.0)	3,217	983 (30.06)	1.20 [1.10, 1.32]	**<0**.**001**	1.12 [1.03, 1.23]	**0**.**012**	1.11 [1.01, 1.21]	**0**.**026**
T3 (>22.0–<24.0)	3,075	1,138 (40.20)	1.69 [1.55, 1.84]	**<0**.**001**	1.41 [1.29, 1.54]	**<0**.**001**	1.38 [1.26, 1.51]	**<0**.**001**
P for trend				**<0**.**001**		**<0**.**001**		**<0**.**001**
Continuous								
Per 1.0 increase	9,583	3,025 (31.42)	1.16 [1.13, 1.19]	**<0**.**001**	1.10 [1.08, 1.13]	**<0**.**001**	1.10 [1.07, 1.12]	**<0**.**001**

BMI, body mass index; T, tertile; HR, hazard ratio; CI, confidence interval; WHR, waist to hip ratio; SBP, systolic blood pressure; DBP, diastolic blood pressure.

**Model 1**: adjusted for sex, age, WHR, SBP, DBP, smoking, and drinking.

**Model 2 (Full model)**: Model 1+ further adjusted for region, urban residence, education, occupation, dietary intake of fat, protein and carbohydrate, and diabetes mellitus.

a Incident rate was presented as per 1,000 person-years of follow-up.

### 3.3 Association between BMI trajectories and new-onset hypertension

**Figure 3:**
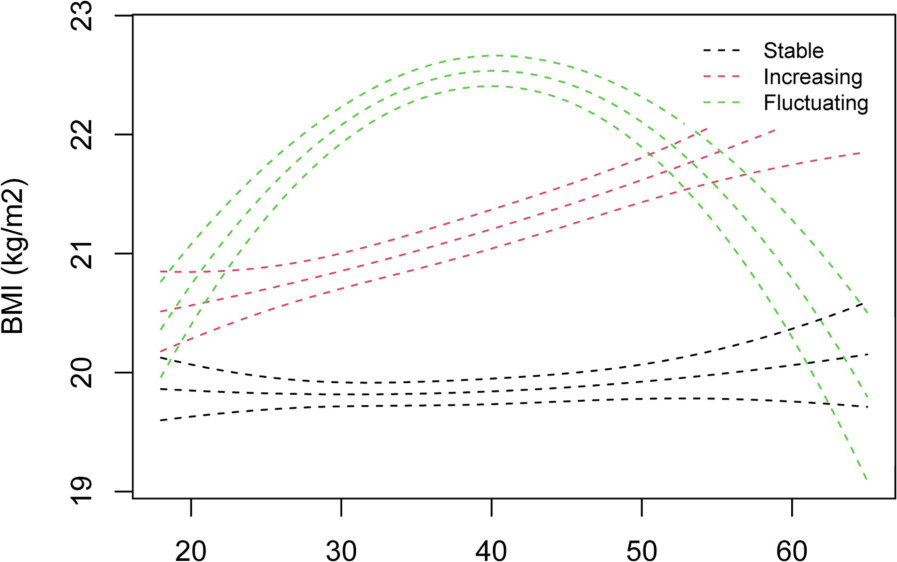
Trajectories of normal BMI among participants aged 18–64 years. BMI, body mass index.

We identified three trajectories of normal BMI in 3,164 participants aged 18–64 years, labeled as stable (*n* = 873), increasing (*n* = 1,390), and fluctuating (*n* = 901) (Figure 3). Compared to stable group, the increasing and fluctuating trajectories were significantly associated with hypertension, respectively (aHR, 1.19, 95% CI, 1.03–1.38, the increasing group; aHR, 1.26, 95% CI, 1.07–1.48, the fluctuating group) (Table 3). The associations remained consistent in 3,322 participants aged ≥18 years (Supplementary Figure S1; Supplementary Table S2).

**Table 3:** The association of BMI trajectories with new-onset hypertension.

BMI trajectories	Total patients	No. of events (incident rate^a^)	Crude model		Model 1		Model 2	
			HR (95% CI)	*P* value	HR (95% CI)	*P* value	HR (95% CI)	*P* value
Stable	873	296 (27.86)	Reference		Reference		Reference	
Increasing	1,390	491 (31.79)	1.21 [1.04, 1.39]	**0.011**	1.22 [1.06, 1.42]	**0**.**007**	1.19 [1.03, 1.38]	**0**.**018**
Fluctuating	901	320 (31.40)	1.18 [1.01, 1.38]	**0.039**	1.31 [1.11, 1.54]	**0**.**001**	1.26 [1.07, 1.48]	**0**.**006**

BMI, body mass index; HR, hazard ratio; CI, confidence interval; WHR, waist to hip ratio; SBP, systolic blood pressure; DBP, diastolic blood pressure.

**Model 1**: adjusted for sex, age, WHR, SBP, DBP, smoking, and drinking.

**Model 2 (Full model)**: Model 1+ further adjusted for region, urban residence, education, occupation, dietary intake of fat, protein and carbohydrate, and diabetes mellitus.

a Incident rate was presented as per 1,000 person-years of follow-up.

### 3.4 Subgroup analyses

**Figure 4:**
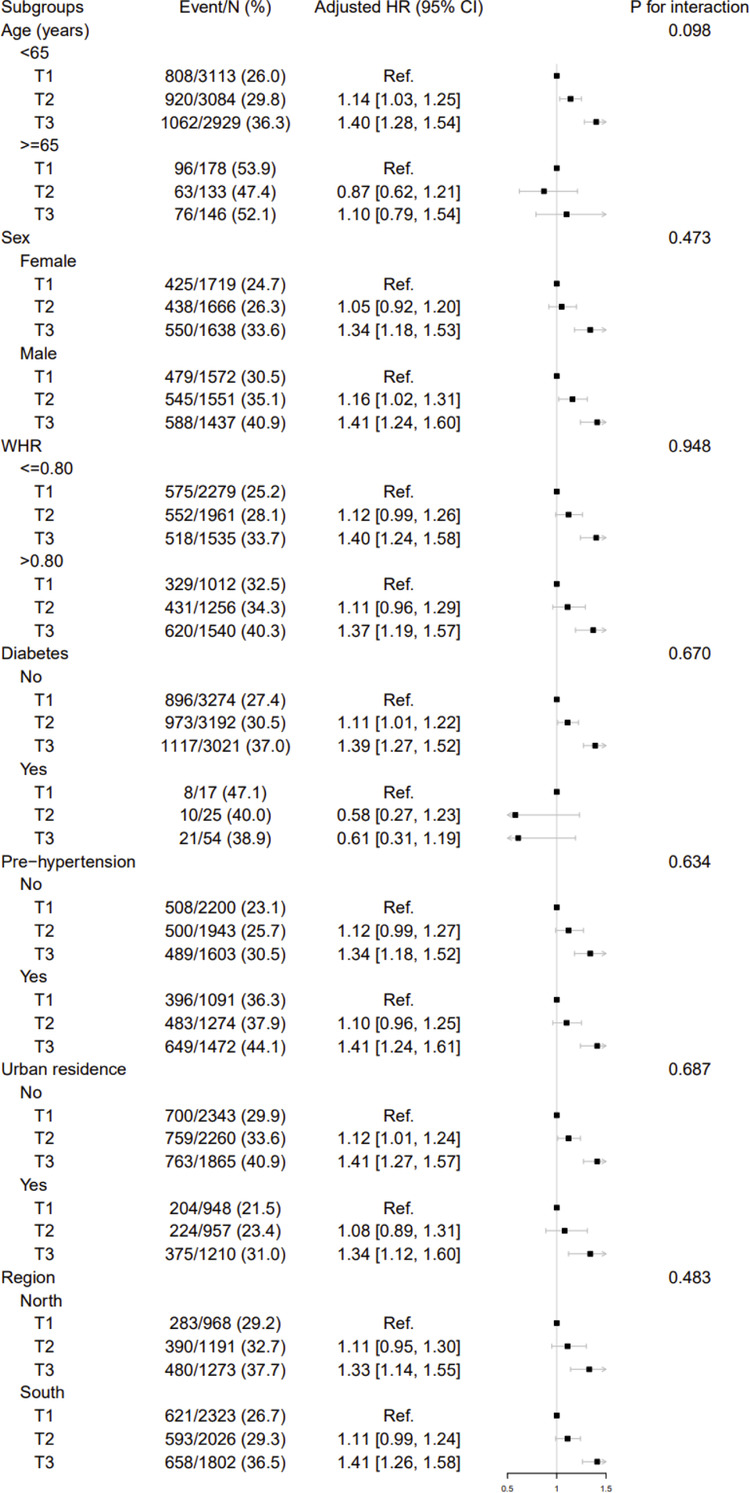
Stratified analyses by potential modifiers of the association between BMI and new-onset hypertension. BMI, body mass index; T, tertile for BMI; HR, hazard ratio; CI, confidence interval; Ref, reference; WHR, waist to hip ratio; SBP, systolic blood pressure; DBP, diastolic blood pressure. The model was adjusted for, if not stratified, sex, age, WHR, SBP, DBP, smoking, drinking, region, urban residence, education, occupation, dietary intake of fat, protein and carbohydrate, and diabetes mellitus.

Stratified analyses were conducted to further explore the association between tertiles of BMI and the risk of new-onset hypertension in various subgroups (Figure 4). None of variables, including age, sex, WHR, urban residence, region, and presence of DM or pre-hypertension, significantly modified this relationship (P for interaction >0.05).

### 3.5 Sensitivity analyses

The results remained consistent when several methods were utilized to verify the robustness of the relationship between BMI and new-onset hypertension (Supplementary Tables S3, S4).

The effect of BMI on new-onset hypertension was consistent when the interval-censoring Cox model was applied for analysis (T2 vs. T1, aHR, 1.11, 95% CI, 1.01–1.20; T3 vs. T1, aHR, 1.38, 95% CI, 1.29–1.47; per 1.0 kg/m^2^ increment, aHR, 1.10, 95% CI, 1.07–1.12). After excluding the questionnaire data used to define hypertension, the results remained largely unchanged (T2 vs. T1, aHR, 1.11, 95% CI, 1.01–1.22; T3 vs. T1, aHR, 1.38, 95% CI, 1.26–1.52; per 1.0 kg/m^2^ increment, aHR, 1.10, 95% CI, 1.07–1.13). Similar trends were observed between BMI and new-onset hypertension after redefining hypertension by the new ACC and AHA guidelines with SBP/DBP threshold of 130/80 (T2 vs. T1, aHR, 1.16, 95% CI, 1.07–1.26; T3 vs. T1, aHR, 1.21, 95% CI, 1.12–1.32; per 1.0 kg/m^2^ increment, aHR, 1.06, 95% CI, 1.04–1.09). Additionally, after redefining the normal-weight participants as those with a BMI between 18.5 kg/m^2^ and 24.9 kg/m^2^ based on the WHO definition, we identified 10,563 non-hypertension participants with normal BMI. The Cox model also showed that high BMI levels were significantly associated with an increased risk of new-onset hypertension (T2 vs. T1, aHR, 1.12, 95% CI, 1.03–1.23; T3 vs. T1, aHR, 1.51, 95% CI, 1.38–1.64; per 1.0 kg/m^2^ increment, aHR, 1.12, 95% CI, 1.10–1.14). Among participants who maintained a normal BMI at all visits, high BMI levels continued to be associated with a higher risk of new-onset hypertension (T2 vs. T1, aHR, 1.07, 95% CI, 0.92–1.24; T3 vs. T1, aHR, 1.20, 95% CI, 1.03–1.40; per 1.0 kg/m^2^ increment, aHR, 1.06, 95% CI, 1.01–1.11) (Supplementary Table S3). Finally, similar trends were found between BMI and new-onset hypertension after further adjusting for the calendar year of enrollment (T2 vs. T1, aHR, 1.10, 95% CI, 1.00–1.21; T3 vs. T1, aHR, 1.35, 95% CI, 1.24–1.48; per 1.0 kg/m^2^ increment, aHR, 1.09, 95% CI, 1.06–1.12) (Supplementary Table S4).

## 4 Discussion

In this large, national, longitudinal cohort study among general Chinese adults, containing 9,583 adults with normal BMI at baseline, we found a positive association between BMI and new-onset hypertension after adjusting for confounders, and this relationship was consistent across various subgroups and in sensitivity analyses. The dose-response curve indicated a positive, linear association between BMI and the risk of new-onset hypertension. Also, we found that increasing and fluctuating BMI trajectories within normal ranges were associated with higher risks of hypertension. Our results support the hypothesis that a high BMI with its increasing and fluctuating BMI trajectory, even when it falls below the threshold for overweight, is associated with increased risks of hypertension. To the best of our knowledge, this is the first national prospective study that strictly limits participants to those with a normal BMI to investigate the relationship between BMI as well as its trajectory and new-onset hypertension. The present study may provide new insights into the primary prevention of hypertension.

Overweight and obesity have been widely recognized as significant factors contributing to hypertension over the past few decades (34). A previous study in China has found that the prevalence of hypertension is approximately 11.2% in adults with normal weight, 20.7% in those who are overweight, and nearly 36.9% in adults who have obesity (35). Various studies have explored the relationship between BMI and the risk of hypertension, consistently showing a higher risk of hypertension in adults who are overweight or obese (5–10). A recent meta-analysis in adults further demonstrated a continuous dose-dependent association between BMI and risk of hypertension (18). However, there is a lack of evidence regarding the impact of BMI within the normal range on development of new-onset hypertension in general adults. Our study found that a high normal BMI, even below the threshold for overweight, is also associated with an increased risk of new-onset hypertension in Chinese adults. These results are consistent with the findings of previous studies conducted among children and adolescents (19, 20). Our study also suggests that maintaining a low normal BMI may be an effective strategy for the primary prevention of hypertension. Notably, our study further demonstrates that, compared to stable BMI trajectory, both steadily increasing and fluctuating trajectories were significantly associated with new-onset hypertension in adults with normal BMI. This finding underscores the importance of long-term maintenance of a stable BMI.

Several studies have demonstrated a gender-specific correlation between BMI and the incidence of hypertension (7–10, 13, 14, 36, 37). A prospective study conducted by Rebecca et al (14) showed that compared to participants in the lowest BMI quintile (<22.4 kg/m^2^), the relative risks of developing hypertension were higher for men with a BMI of 22.4–23.6, 23.7–24.7, 24.8–26.4, and >26.4 kg/m^2^, founding a strong gradient between higher BMI and increased risk of hypertension, even within the “normal” and mildly “overweight” BMI range. Similarly, in female participants, higher BMI was significantly associated with the development of hypertension compared with the lowest BMI, even within the high normal BMI group (7, 36). In both male and female participants, overweight and obesity were significantly associated with the development of hypertension compared with the normal reference group (8, 9, 37). Our subgroup analyses showed that a high normal BMI was significantly associated with the development of hypertension compared to low normal BMI, regardless of gender. These findings align with the previous studies (10, 13), which emphasizes that maintaining a low normal BMI may be recommended for both non-hypertensive men and women to prevention of incident hypertension.

The exact mechanism by which stable adiposity raises blood pressure remains uncertain, though several possible mechanisms including abnormal activity of the sympathetic nervous system and renin-angiotensin-aldosterone system (RAAS) have been proposed (38–41). Weight increase may trigger complex physiological and biochemical processes in the body, such as decreased insulin sensitivity and the development of insulin resistance (IR). Our previous study demonstrated that a positive association between triglyceride-glucose (TyG) index (a surrogate of IR) and new-onset hypertension (27), further supporting these hypotheses. In addition, inflammatory pathways may play an important role in this association (42–45). On the one hand, weight gain can lead to increased release of adipokines (e.g., leptin, tumor necrosis factor alpha, retinol-binding protein 4) and cytokines from adipose tissue, leading to inflammatory endothelial dysfunction and metabolic dysfunction (46, 47). On the other hand, adipocytokines may increase peripheral vascular resistance, potentially raising blood pressure by overstimulating the aforementioned RASS pathway (48, 49).

This study has several strengths. First, one of the major strengths of this study is the use of the long-term national, longitudinal population-based cohort with a large number of subjects, which allowed us to thoroughly explore the relationship between BMI within normal range and new-onset hypertension. Second, we adjusted for important potential confounders, such as baseline blood pressure, WHR, dietary intakes, and the history of diabetes, to enhance the accuracy of our findings. Third, our study included subgroup and sensitivity analyses, which confirmed the stability of the results.

Nevertheless, certain limitations also existed in this study. First, although we adjusted for potential confounders as comprehensively as possible, some unmeasured confounders may remain and residual confounding could not be entirely ruled out. For example, because body fat percentage was not measured in the CHNS population, the study model did not adjust for this factor. Second, limited by the nature of observational studies, we could not determine the causal relationship between high normal BMI and new-onset hypertension. However, the prospective study design does strengthen the level of evidence. Third, the definition of hypertension was based on physician on-site blood pressure measurement data and questionnaires, which may introduce recall bias. Nevertheless, the findings remained consistent even after excluding questionnaire-based information on hypertension obtained in the sensitivity analysis. Last, this study was limited to the Chinese general population, and further research is needed to confirm the consistency of these findings in other ethnic and national populations.

In conclusion, our study demonstrated that a high and fluctuating BMI within normal ranges is significantly associated with an increased risk of new-onset hypertension among Chinese adults. Our findings suggest that maintaining a relatively low normal BMI with stable trajectory in non-hypertensive individuals with normal weight might help with the primary prevention of hypertension.

## Data Availability

Publicly available datasets were analyzed in this study. This data can be found here: China Health and Nutrition Survey repository [http://www.cpc.unc.edu/projects/china].
