# Supplementary material for: Effect of high normal body mass index and its trajectory on risk of new-onset hypertension among Chinese adults: a national prospective cohort study
Source: Front Cardiovasc Med. 2025 Nov 21;12:1684124. doi: 10.3389/fcvm.2025.1684124 (PMC12679297; doi:10.3389/fcvm.2025.1684124)
Supplement: Supplementary file 1 [file Datasheet1.docx]

**Supplemental Ma****terial**

**Supplemental Tables**

**Table S1.** Baseline characteristics of participants stratified by outcome of hypertension.

**Table S2.** The association between BMI trajectories and new-onset hypertension in participants aged ≥18 years.

**Table S3.** Sensitivity analyses of the association between BMI and new-onset hypertension.

**Table S4.** The association between BMI and new-onset hypertension adjusted for the calendar year of enrollment.

**Supplemental Figures**

**Figure S1.** Trajectories of normal BMI among participants aged ≥18 years.

This supplementary material has been provided by the authors to give readers additional information about their work.

**Table S1.** Baseline characteristics of participants stratified by outcome of hypertension.

| **Characteristics** | **Overall** | **Outcome of hypertension** | | |
| --- | --- | --- | --- | --- |
|  |  | **No** | **Yes** | ***P* value** |
| n | 9583 | 6558 | 3025 |  |
| BMI, kg/m² | 21.2 [20.0, 22.5] | 21.1 [19.9, 22.3] | 21.5 [20.2, 22.7] | **<0.001** |
| Age, years | 36.0 [27.0, 47.0] | 33.0 [25.0, 43.0] | 42.0 [34.0, 53.0] | **<0.001** |
| Male (%) | 4560 (47.6) | 2948 (45.0) | 1612 (53.3) | **<0.001** |
| SBP, mmHg | 110.0 [103.0, 120.0] | 110.0 [101.0, 120.0] | 115.0 [108.0, 120.0] | **<0.001** |
| DBP, mmHg | 73.0 [69.0, 80.0] | 71.0 [68.0, 79.0] | 75.0 [70.0, 80.0] | **<0.001** |
| WHR | 0.8 [0.8, 0.9] | 0.8 [0.8, 0.9] | 0.8 [0.8, 0.9] | **<0.001** |
| Smoking, n (%) |  |  |  | **<0.001** |
| never | 6536 (68.2) | 4636 (70.7) | 1900 (62.8) |  |
| ever | 123 (1.3) | 78 (1.2) | 45 (1.5) |  |
| current | 2924 (30.5) | 1844 (28.1) | 1080 (35.7) |  |
| Drinking, n (%) |  |  |  | **<0.001** |
| never | 6315 (65.9) | 4460 (68.0) | 1855 (61.3) |  |
| ever | 113 (1.2) | 64 (1.0) | 49 (1.6) |  |
| current | 3155 (32.9) | 2034 (31.0) | 1121 (37.1) |  |
| Urban residence, n (%) | 3115 (32.5) | 2312 (35.3) | 803 (26.5) | **<0.001** |
| Region^*^, n (%) |  |  |  | **0.002** |
| North | 3432 (35.8) | 2279 (34.8) | 1153 (38.1) |  |
| South | 6151 (64.2) | 4279 (65.2) | 1872 (61.9) |  |
| Education, n (%) |  |  |  | **<0.001** |
| Illiteracy | 1844 (19.2) | 907 (13.8) | 937 (31.0) |  |
| Primary school | 1904 (19.9) | 1199 (18.3) | 705 (23.3) |  |
| Middle school | 4619 (48.2) | 3461 (52.8) | 1158 (38.3) |  |
| High school or above | 1216 (12.7) | 991 (15.1) | 225 (7.4) |  |
| Occupation, n (%) |  |  |  | **<0.001** |
| Farmer | 3802 (39.7) | 2280 (34.8) | 1522 (50.3) |  |
| Worker | 3157 (32.9) | 2400 (36.6) | 757 (25.0) |  |
| Unemployed | 2207 (23.0) | 1564 (23.8) | 643 (21.3) |  |
| Others | 417 (4.4) | 314 (4.8) | 103 (3.4) |  |
| Dietary intake, g/d |  |  |  |  |
| Energy | 2259.1 [1825.7, 2709.9] | 2203.8 [1773.4, 2649.1] | 2355.0 [1940.4, 2825.9] | **<0.001** |
| Fat | 60.9 [41.1, 85.9] | 62.3 [41.8, 87.4] | 58.8 [40.0, 82.4] | **<0.001** |
| Carbohydrate | 338.0 [251.0, 429.8] | 325.2 [240.2, 413.3] | 366.7 [286.0, 461.6] | **<0.001** |
| Protein | 67.1 [54.1, 83.3] | 66.5 [53.6, 82.7] | 68.6 [55.4, 84.8] | **<0.001** |
| Diabetes mellitus, n (%) | 96 (1.0) | 57 (0.9) | 39 (1.3) | 0.070 |

Results are expressed as median [interquartile range] or number (percentage).

^*^Region was divided into north (Heilongjiang, Liaoning, Shandong, and Henan), and south (Jiangsu, Hubei, Hunan, Guizhou, and Guangxi) based on the Qinling Mountains-Huaihe River Line.

**Abbreviations**: n, number; BMI, body mass index; SBP, systolic blood pressure; DBP, diastolic blood pressure; WHR, waist to hip ratio.

**Table S2.** The association between BMI trajectories and new-onset hypertension in participants aged ≥18 years.

| **BMI trajectories** | **Total patients** | **No. of Events**  **(incident rate^a^)** | **Crude model** | | **Model 1** | | **Model 2** | |
| --- | --- | --- | --- | --- | --- | --- | --- | --- |
|  |  |  | **HR (95% CI)** | ***P* value** | **HR (95% CI)** | ***P* value** | **HR (95% CI)** | ***P* value** |
| Stable | 1,005 | 358 (30.34) | Reference |  | Reference |  | Reference |  |
| Increasing | 1,417 | 504 (32.68) | 1.12 [0.98, 1.29] | 0.092 | 1.17 [1.02, 1.35] | **0.021** | 1.15 [1.00, 1.32] | **0.046** |
| Fluctuating | 900 | 329 (32.74) | 1.11 [0.96, 1.29] | 0.167 | 1.26 [1.08, 1.47] | **0.003** | 1.23 [1.05, 1.43] | **0.010** |

**Abbreviations**: BMI, body mass index; HR, hazard ratio; CI, confidence interval; WHR, waist to hip ratio; SBP, systolic blood pressure; DBP, diastolic blood pressure.

^a^Incident rate was presented as per 1000 person-years of follow-up.

**Model 1**: adjusted for sex, age, WHR, SBP, DBP, smoking, and drinking.

**Model 2 (Full model)**: Model 1+further adjusted for region, urban residence, education, occupation, dietary intake of fat, protein and carbohydrate, and diabetes mellitus.

**Table S3.** Sensitivity analyses of the association between BMI and new-onset hypertension.

| **BMI (kg/m^2^)** | **Total patients** | **No. of Events**  **(incident rate^a^)** | **Crude model** | | **Model 1** | | **Model 2** | |
| --- | --- | --- | --- | --- | --- | --- | --- | --- |
|  |  |  | **HR (95% CI)** | ***P* value** | **HR (95% CI)** | ***P* value** | **HR (95% CI)** | ***P* value** |
| **Cox model using interval-censoring methods (n=9583)** | | | | | | | | |
| Tertiles |  |  |  |  |  |  |  |  |
| T1 (18.5 to 20.4) | 3,291 | 904 (25.64) | Reference |  | Reference |  | Reference |  |
| T2 (>20.4 to 22.0) | 3,217 | 983 (30.06) | 1.19 [1.10, 1.28] | **<0.001** | 1.12 [1.02, 1.21] | **0.021** | 1.11 [1.01, 1.20] | **0.033** |
| T3 (>22.0 to <24.0) | 3,075 | 1,138 (40.20) | 1.64 [1.56, 1.73] | **<0.001** | 1.38 [1.47, 1.29] | **<0.001** | 1.38 [1.29, 1.47] | **<0.001** |
| P for trend |  |  |  | **<0.001** |  | **<0.001** |  | **<0.001** |
| Continuous |  |  |  |  |  |  |  |  |
| Per 1.0 increase | 9,583 | 3,025 (31.42) | 1.15 [1.13, 1.18] | **<0.001** | 1.10 [1.07, 1.12] | **<0.001** | 1.10 [1.07, 1.12] | **<0.001** |
| **Hypertension diagnosed only by blood pressure data (n=9774)** | | | | | | | | |
| Tertiles |  |  |  |  |  |  |  |  |
| T1 (18.5 to 20.4) | 3,329 | 871 (24.37) | Reference |  | Reference |  | Reference |  |
| T2 (>20.4 to 22.0) | 3,275 | 960 (28.86) | 1.22 [1.11, 1.33] | **<0.001** | 1.12 [1.02, 1.23] | **0.014** | 1.11 [1.01, 1.22] | **0.028** |
| T3 (>22.0 to <24.0) | 3,170 | 1,122 (38.58) | 1.71 [1.56, 1.86] | **<0.001** | 1.40 [1.28, 1.53] | **<0.001** | 1.38 [1.26, 1.52] | **<0.001** |
| P for trend |  |  |  | **<0.001** |  | **<0.001** |  | **<0.001** |
| Continuous |  |  |  |  |  |  |  |  |
| Per 1.0 increase | 9,774 | 2,953 (30.11) | 1.17 [1.14, 1.20] | **<0.001** | 1.10 [1.08, 1.13] | **<0.001** | 1.10 [1.07, 1.13] | **<0.001** |
| **Hypertension redefined as an average SBP ≥130 mmHg and/or an average DBP ≥80 mmHg, a physician hypertension diagnosis, or taking anti-hypertension medication (n=5659)** | | | | | | | | |
| Tertiles |  |  |  |  |  |  |  |  |
| T1 (18.5 to 20.3) | 1,990 | 1,103 (68.24) | Reference |  | Reference |  | Reference |  |
| T2 (>20.3 to 21.8) | 1,828 | 1,115 (81.13) | 1.23 [1.13, 1.34] | **<0.001** | 1.19 [1.09, 1.29] | **<0.001** | 1.16 [1.07, 1.26] | **<0.001** |
| T3 (>21.8 to <24.0) | 1,841 | 1,197 (91.06) | 1.42 [1.30, 1.54] | **<0.001** | 1.27 [1.17, 1.38] | **<0.001** | 1.21 [1.12, 1.32] | **<0.001** |
| P for trend |  |  |  | **<0.001** |  | **<0.001** |  | **<0.001** |
| Continuous |  |  |  |  |  |  |  |  |
| Per 1.0 increase | 5,659 | 3,415 (79.32) | 1.11 [1.08, 1.13] | **<0.001** | 1.08 [1.05, 1.10] | **<0.001** | 1.06 [1.04, 1.09] | **<0.001** |
| **Normal BMI redefined as BMI ≥18.5 kg/m^2^ and BMI <25.0 kg/m^2^ (n=10563)** | | | | | | | | |
| Tertiles |  |  |  |  |  |  |  |  |
| T1 (18.5 to 20.6) | 3,683 | 1,017 (25.88) | Reference |  | Reference |  | Reference |  |
| T2 (>20.6 to 22.4) | 3,414 | 1,082 (31.38) | 1.25 [1.14, 1.36] | **<0.001** | 1.14 [1.05, 1.24] | **0.003** | 1.12 [1.03, 1.23] | **0.008** |
| T3 (>22.4 to <25.0) | 3,466 | 1,339 (44.21) | 1.89 [1.74, 2.05] | **<0.001** | 1.54 [1.41, 1.67] | **<0.001** | 1.51 [1.38, 1.64] | **<0.001** |
| P for trend |  |  |  | **<0.001** |  | **<0.001** |  | **<0.001** |
| Continuous |  |  |  |  |  |  |  |  |
| Per 1.0 increase | 10,563 | 3,438 (33.03) | 1.19 [1.16, 1.21] | **<0.001** | 1.13 [1.10, 1.15] | **<0.001** | 1.12 [1.10, 1.14] | **<0.001** |
| **Adults with stable normal BMI (n=4718)** | | | | | | | | |
| Tertiles |  |  |  |  |  |  |  |  |
| T1 (18.5 to 20.3) | 1,695 | 356 (21.72) | Reference |  | Reference |  | Reference |  |
| T2 (>20.3 to 21.6) | 1,533 | 340 (25.05) | 1.20 [1.04, 1.40] | **0.015** | 1.07 [0.92, 1.24] | 0.408 | 1.07 [0.92, 1.24] | 0.410 |
| T3 (>21.6 to <24.0) | 1,490 | 345 (31.66) | 1.63 [1.40, 1.89] | **<0.001** | 1.22 [1.05, 1.42] | **0.011** | 1.20 [1.03, 1.40] | **0.019** |
| P for trend |  |  |  | **<0.001** |  | **0.012** |  | **0.019** |
| Continuous |  |  |  |  |  |  |  |  |
| Per 1.0 increase | 4,718 | 1,041 (25.48) | 1.17 [1.12, 1.23] | **<0.001** | 1.06 [1.01, 1.12] | **0.010** | 1.06 [1.01, 1.11] | **0.021** |

**Abbreviations**: BMI, body mass index; n, number; T, tertile for BMI; HR, hazard ratio; CI, confidence interval; WHR, waist to hip ratio; SBP, systolic blood pressure; DBP, diastolic blood pressure.

^a^Incident rate was presented as per 1000 person-years of follow-up.

**Model 1**: adjusted for sex, age, WHR, SBP, DBP, smoking, and drinking.

**Model 2 (Full model)**: Model 1+further adjusted for region, urban residence, education, occupation, dietary intake of fat, protein and carbohydrate, and diabetes mellitus.

**Table S4.** The association between BMI and new-onset hypertension adjusted for the calendar year of enrollment.

| **BMI (kg/m^2^)** | **Total patients** | **No. of Events**  **(incident rate^a^)** | **Crude model** | | **Model 1** | |
| --- | --- | --- | --- | --- | --- | --- |
|  |  |  | **HR (95% CI)** | ***P* value** | **HR (95% CI)** | ***P* value** |
| Tertiles |  |  |  |  |  |  |
| T1 (18.5 to 20.4) | 3,291 | 904 (25.64) | Reference |  | Reference |  |
| T2 (>20.4 to 22.0) | 3,217 | 983 (30.06) | 1.20 [1.10, 1.32] | **<0.001** | 1.10 [1.00, 1.21] | **0.039** |
| T3 (>22.0 to <24.0) | 3,075 | 1,138 (40.20) | 1.69 [1.55, 1.84] | **<0.001** | 1.35 [1.24, 1.48] | **<0.001** |
| P for trend |  |  |  | **<0.001** |  | **<0.001** |
| Continuous |  |  |  |  |  |  |
| Per 1.0 increase | 9,583 | 3,025 (31.42) | 1.16 [1.13, 1.19] | **<0.001** | 1.09 [1.06, 1.12] | **<0.001** |

Abbreviations: BMI, body mass index; T, tertile; HR, hazard ratio; CI, confidence interval; WHR, waist to hip ratio; SBP, systolic blood pressure; DBP, diastolic blood pressure.

^a^Incident rate was presented as per 1000 person-years of follow-up.

Model 1: adjusted for sex, age, WHR, SBP, DBP, smoking, drinking, region, urban residence, education, occupation, dietary intake of fat, protein and carbohydrate, diabetes mellitus, and calendar year of enrollment.


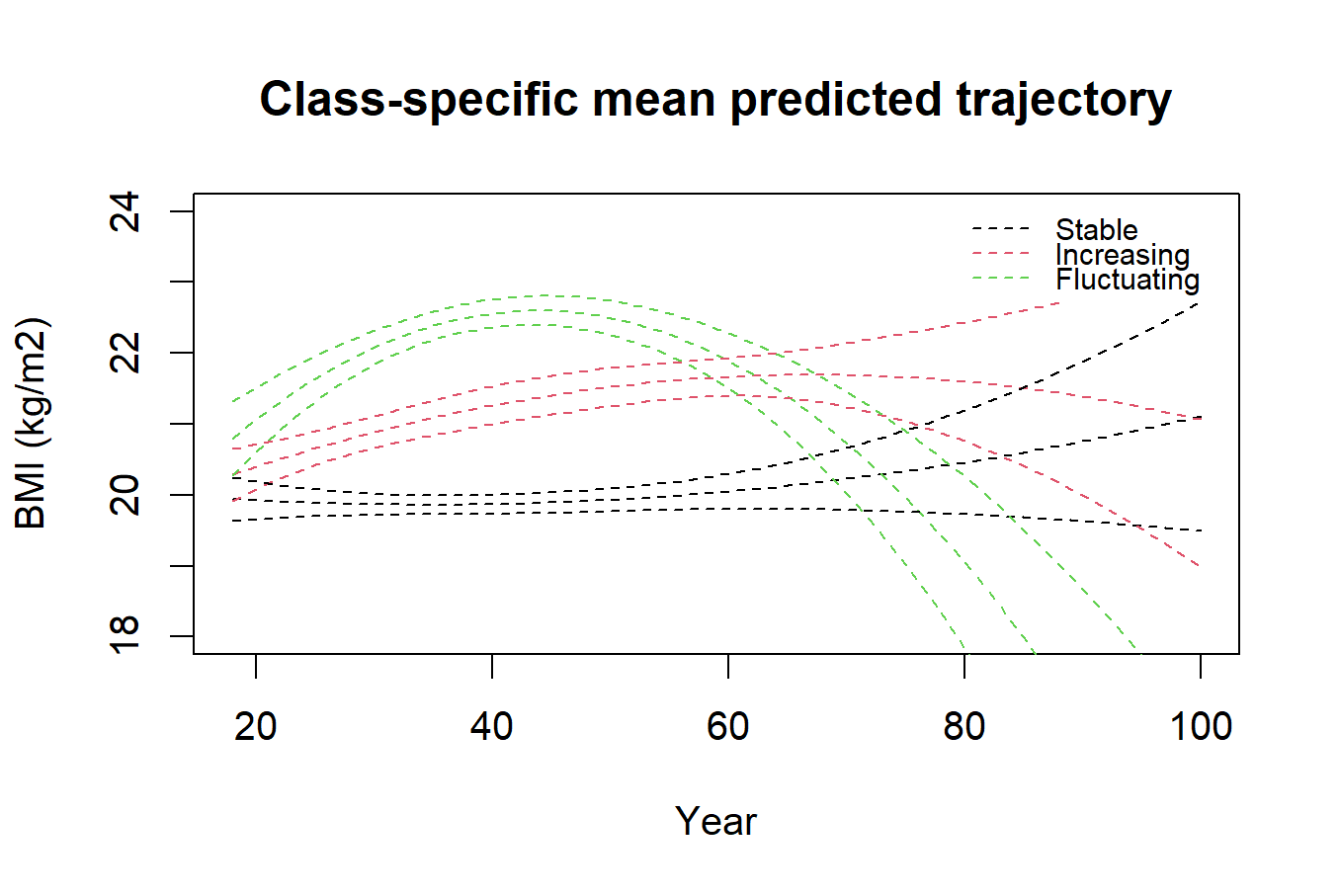


**Figure S1.** Trajectories of normal BMI among participants aged ≥18 years.

**Abbreviations:** BMI, body mass index.
